# Understanding Current Needs and Future Expectations of Informal Caregivers for Technology to Support Health and Well-being: National Survey Study

**DOI:** 10.2196/15413

**Published:** 2022-01-27

**Authors:** Kieren J Egan, Patricia Clark, Zahid Deen, Carmen Paputa Dutu, Graham Wilson, Lisa McCann, Marilyn Lennon, Roma Maguire

**Affiliations:** 1 Digital Health and Wellness Group Department of Computing and Information Sciences University of Strathclyde Glasgow United Kingdom; 2 Carers Scotland Glasgow United Kingdom; 3 The Health and Social Care Alliance Scotland Glasgow United Kingdom

**Keywords:** caregiving, technology, health, well-being, digital health, co-design, mobile phone

## Abstract

**Background:**

There are approximately 6.5 million informal (unpaid) caregivers in the United Kingdom. Each caregiver plays a critical role in the society, supporting the health and well-being of those who are ill, disabled, or older and who need frequent support. Digital technologies are becoming a ubiquitous part of everyday life for many, but little is known about the *real-world* impact of technology for those in a caring role, including the abilities of technologies to address the mental and physical impacts of caregiving.

**Objective:**

This study aims to understand the current and future technology use of caregivers, including digital technologies used to care for themselves and the person they look after.

**Methods:**

We codeveloped a wide range of questions with caregivers and care professionals and delivered this survey both on the web and in paper format (eg, using social networks such as Twitter alongside *in-person* events). Questions were focused on providing care and looking after caregiver health and well-being. Analyses focused on both quantitative outcomes (frequency counts and Likert questions) and explored free text entries (thematic analysis).

**Results:**

From 356 respondents, we identified that caregivers were receptive to, and largely positive about current and future use of technology both for their own care and their caring role (eg, checking in from distance). There were notable concerns, including the risk that technology could replace human contact. We identified several key areas for future work, including communication with health and social care professionals, and the potential for technology to help caregivers with their own health. We also identified several stakeholders (eg, care workers, pharmacy staff, and general practitioners) who could act as suitable points for technology signposting and support.

**Conclusions:**

Caregivers are a transient, often difficult to reach population, and this work has collated a large body of knowledge across a diverse group of individuals. Many caregivers, like the rest of society, are realizing the benefits of using everyday technology to help deliver care. It is clear that there is already a high level of dependency on technologies, where future expectations will grow. However, many barriers to digital technology use remain, including a lack of ongoing technology support. Preventive measures linked to technology that can help look after a caregiver’s own health appear acceptable, particularly for communicative tools. This collated caregiver knowledge is a call for all stakeholders—academics, policy makers, and practitioners—to take note of these specific challenges, and to ensure that caregiver voices are both heard and fully integrated within the emerging digital health agenda.

## Introduction

### Background

Informal (unpaid) caregivers play a critical role in society, supporting the health and well-being of those who are ill, disabled, or older who need frequent support. Caregivers are the biggest health care provider in the United Kingdom alone, with an estimated 6.5 million informal caregivers (ie, approximately 10% of the total population), with an economic impact of approximately US $77-US $182 billion per year [1,2]. Globally, an aging and growing population means that the number and economic contribution of caregivers appear to grow considerably in the years ahead.

Collectively, caregivers are diverse. Each situation is unique, varying across geographic location, conditions cared for, cumulative time spent caring, available support (social, health care, or otherwise), and information received [3]. Reaching and understanding this *silent workforce* is not straight forward, as many caregivers take many years to—or may never—identify with the term of being a *caregiver* [4]. Although the tasks that caregivers undertake differ (eg, specific needs, hours spent caregiving, and support available [5-7]), the commonalities in experience are the physical and mental stresses, which are considerable and unrelenting. The cumulative load of caregiving steadily impacts the health and well-being of caregivers, and for many, caregiving is associated with a broad range of acute and chronic mental and physical conditions [8-10]. Accordingly, there are urgent calls at national and multinational levels to find ways to support caregiver needs through cost-effective, sustainable, and preventive solutions [11,12]. Such calls have accelerated the development of many innovative approaches, such as those directed toward digital health and wellness technology-based solutions [13].

The development of solutions based on digital health and wellness technology is increasing across a wide range of approaches, including telehealth, mobile health, wearables, and health analytics as well as digitalized (eg, paperless) health systems [14,15]. Technology support for health and well-being is also increasingly prominent within community care where technologies, such as webcams, personal alarms, GPS trackers, and voice technologies are helping many caregivers regularly manage aspects of safety, communication, care, and sustaining independence for as long as possible [13].

### Study Aims

In a growing continuum of digital possibilities, understanding theoretical models of moderators and mediators for technology use is of significant interest to all stakeholders [16]. Nevertheless, our global understanding of the barriers and enablers for *real-world* technology use for caregivers is still surprisingly sparse [17-19]. Given the considerable prominence of caregivers within our health and social care workforce, this study aims to collect evidence on the current and future technology use of caregivers, both for their own health and well-being, and the person or persons they look after within the context of the United Kingdom.

## Methods

### Overview

The Supported Carer Project survey involved delivering a comprehensive survey to help inform current understanding of the use of digital technology among caregivers, both for caregiver health and those cared for. As we did not find one already in existence, we co-designed a novel survey with caregivers. This survey was designed to be used across the United Kingdom to capture both current and future needs and how digital health technologies might be able to meet these needs.

### Inclusion Criteria and Survey Co-design

Our inclusion criteria reflected our broad interests and included all informal caregivers aged ≥18 years. We used a broad definition of an informal caregiver, *people that provide unpaid care by looking after an ill, older or disabled family member, friend, or partner*. We did not specify the minimum number of hours per week caregivers needed to be caring for. Ethical approval was received from the Department of Computer & Information Science at the University of Strathclyde. Our survey collated information on basic demographics, health needs, and explored perspectives on technology for both caregivers and those being cared for (including interactions with health and social care professionals). Our survey questions (Multimedia Appendix 1) were codeveloped through consultation with key study partners who had significant experience working in the caregiver domain (Health and Social Care Alliance Scotland [The ALLIANCE] and Carers Scotland as part of Carers UK). Questions were developed iteratively, and the scope and approach of our survey was critiqued by 3 individual caregivers and 5 professionals from caregiver organizations to ensure that the length, wording, and scope were appropriate. No questions were mandatory, so responses vary across each question discussed. The survey consisted of four key sections: (1) the demographic details of you as a caregiver (7 questions); (2) you as a caregiver and technology use (13 questions); (3) the demographic details of the person that you care for and health and social care service use (26 questions); and (4) technology use for the person you care for (50 questions). Caregivers could add multiple people cared for should they care for more than one person, up to a maximum of 4 people.

### Survey Distribution and Consent

The distribution of our survey involved convenience sampling. More specifically, we shared the web-based version of our survey through social media channels (eg, Twitter), and email distribution through networks accessible to our third sector partners. The survey was promoted using email and social media networks connected to The ALLIANCE [20] and Caregivers Scotland as part of the Caregivers United Kingdom [21]. Paper copies of the survey were also distributed at conferences and professional events from The ALLIANCE and Carers UK (Scotland), which were posted back to us. In addition, the UK Alzheimer’s Society agreed to share this survey using their web-based message board systems, *Talking Point*. The survey was available from June 21, 2018, to September 28, 2018. Consent was implied in both digital and paper format after participants read, acknowledged, and accepted the initial terms of the anonymized survey.

### Data Handling and Analyses

Our survey was constructed using Qualtrics Software, where we stated that any data entered must not contain any identifiable information. These efforts were paralleled in a paper format. As free text entry methods could not prevent identifiable information from being entered, data were treated as confidential at all times and retained within encrypted, password-protected sources. Qualitative analyses were performed using thematic analysis and deep dives [22]. All quantitative analyses (frequencies and summary statistics) were performed using R Studio (version 1.1.456).

## Results

### Demographic Information of Caregivers and Those Cared for

We received 356 caregiver responses (total sample size but individual question responses vary) in our survey, and the demographics of caregivers and those cared for are summarized in Table 1. Caregivers who completed our survey varied in age, but most commonly between 45 and 54 years (135/356, 37.9% of responses), predominantly female (288/354, 81.3% vs 60/354, 16.9%) and self-reported as White (335/354, 94.6%). Of the 331 responses for this specific question, 234 (70.7%) of our respondents were located in Scotland, 74 (22.3%) in England, 17 (5.1%) in Wales, and 6 (1.8%) in Northern Ireland. Regarding the highest level of education, 56.2% (200/356) of respondents had obtained a degree or equivalent, and 22.2% (79/356) had completed higher education. Regarding the number of people cared for, 72.9% (210/288) of participants were caring for 1 person, 22.2% (64/288) were caring for 2 people, 4.2% (12/288) were caring for 3 people, and 0.7% (2/288) were caring for 4 or more people. A total of 34.2% (121/354) of caregivers were working full-time, 25.4% (90/354) were part-time, and 40.4% (143/354) were not working, of which 90 (ie, 90/354, 25.4% of all caregivers responding) had to give up work because of caregiving. Caregivers varied in the number of years spent caregiving, ranging from less than a year (5/309, 1.6%) to over 20 years (34/309, 11%). Four participants responded to our survey via post (4/356, 1.1%) with all other responses via our web link.

Our survey responses included information from 359 individuals cared for, where information differed considerably from that among caregivers in both age and gender (Table 2). Those cared for were most commonly either <35 years (99/359, 27.6%) or >65 years (193/359, 53.8%), and males and females cared for were 51.1% (181/354) and 47.7% (169/354), respectively. The ethnicity of those cared for was very similar to that of caregivers. Among individuals cared for (where sufficient detail was given for 355 individuals; Multimedia Appendix 2) over 20 different conditions were listed, and the most common conditions were dementia (109/355, 30.7%), older needs (106/355, 29.8%), and mental health conditions (74/355, 20.8%). Similarly, the types of specific health problems reported varied considerably across those cared for. From 258 responses, 99 (38.4%) reported precise hand movement problems, 66 (25.6%) speech impairments, 66 (25.6%) deafness or hearing loss, and 40 (15.5%) were blind or had sight loss. A total of 44.2% (114/258) of responses indicated that there were *other* sensory issues. We were able to explore 106 of these (free text responses), where mobility was a specific problem for 29.2% (31/106) of respondents. Overall, this subgroup of responses was diverse and problems related to both physical (eg, “nerve damage” and “physically weak”) and psychosocial health issues (eg, anxiety or memory).

**Table 1:** Study sample and population characteristics of caregivers.

Demographics			Caregivers, n (%)
**Age (years; N=356)**			
	18-24	4 (1.1)	
	25-34	17 (4.8)	
	35-44	56 (15.7)	
	45-54	135 (37.9)	
	55-64	100 (28.1)	
	65-74	28 (7.9)	
	75-84	13 (3.7)	
	≥85	1 (0.3)	
	Prefer not to say	2 (0.6)	
**Gender (n=354)**			
	Female	288 (81.4)	
	Male	60 (16.9)	
	Other	3 (0.85)	
	Prefer not to say	3 (0.85)	
**Ethnicity (n=354)**			
	White	335 (94.6)	
	Mixed/multiple ethnic groups	3 (0.85)	
	Asian/Asian British	4 (1.13)	
	Black/African/Caribbean/Black British	0 (0)	
	Other	4 (1.12)	
	Prefer not to say	8 (2.3)	
**Highest level of education (N=356)**			
	Degree or equivalent	200 (56.2)	
	Higher education	79 (22.2)	
	Other qualifications	18 (5.1)	
	School qualifications	52 (14.6)	
	No qualifications	5 (1.4)	
	Do not know	2 (0.6)	
**Number of years caregiving (n=309)**			
	<1	5 (1.6)	
	1-2	36 (12)	
	3-4	54 (17.5)	
	5-6	53 (17.2)	
	7-8	28 (9.1)	
	9-10	24 (7.8)	
	10-16	41 (13.3)	
	>16-20	34 (11)	
	>20	34 (11)	

**Table 2:** Study sample and population characteristics of people cared for.

Characteristics			People cared for, n (%)
**Age (years; N=359)**			
	0-15	49 (13.7)	
	16-24	32 (8.9)	
	25-34	18 (5)	
	35-44	9 (2.5)	
	45-54	25 (7)	
	55-64	31 (8.7)	
	65-74	39 (10.9)	
	75-84	81 (22.6)	
	≥85	73 (20.3)	
	Prefer not to say	2 (0.6)	
**Gender (n=354)**			
	Female	169 (47.5)	
	Male	181 (50.8)	
	Other	1 (0.3)	
	Prefer not to say	5 (1.5)	
**Ethnicity (n=356)**			
	White	334 (93.8)	
	Mixed/multiple	7 (2)	
	Asian/Asian British	5 (1.4)	
	Mixed/multiple	1 (0.3)	
	Black/African/Caribbean/Black British	2 (0.6)	
	Other	7 (2)	

### Technology for Caregivers’ Own Health and Well-being

#### Current Interest

Caregivers were asked about their level of agreement to use technology to help with their own health and well-being. Of the 277 responses, 92 (33.2%) of respondents strongly agreed, 102 (36.8%) agreed, 67 (24.2%) neither agreed nor disagreed, 12 (4.3%) disagreed, and 4 (1.4%) strongly disagreed:

Being able to access support or some form of mental health support would be ideal. Being a carer is tough and you focus most of your time on the person you care for but forget you also need care.

Participant quote on using technology for own health and well-being

Caregivers interpreted help from digital technologies in many different forms. Our analysis identified themes across concepts of relaxation, meditation, memory prompts, communication (both with health and social care professionals and peers), entertainment and tools, such as fitness trackers to encourage or inform healthy lifestyle choices (Textbox 1). Arguments to support the use of technology to support health and wellness were based on convenience, accessibility, and accuracy: being able to use digital tools quickly to find answers on a regular basis. Exploring the free text of caregivers who were not interested in using technology for their own health (ie, 16 caregivers who disagreed or strongly disagreed that they had an interest) highlighted many concerns regarding technology. Time (and money) were barriers to use for 5 respondents, and technologies that operate in silos outside of health and social care are of limited use. Concerns were also raised that technology can become a *gimmick*. Technologies were highlighted as a concern where they reinforce a concept of *failure*: technologies that assess progress and activity relating to one’s own health and well-being can resonate with feelings of a lack of achievement.

**Caregiver quotes for using technology for own health and well-being. Caregivers rated their agreeability regarding their level of interest “to use technology to help me with my own health and well-being.” Quotes are examples of further details given from participants grouped according to level of agreement.**

**Strongly agree**

- “Being able to access support or some form of mental health support would be ideal. Being a caregiver is tough and you focus most of your time on the person you care for but forget you also need care.”

**Agree**

- “It is all very useful- but won’t encourage you to meditate or exercise. You have to want to do it, for it to be effective. When you are isolated, depressed and stressed—you still need human interaction.”

**Neither agree nor disagree**

- “I don’t know how it would help. I fear it going wrong.”

**Disagree**

- “Gimmicks like these tech devices are of little interest to me. I do make extensive use of the internet to connect with other caregivers and share information and social chatter.”

**Strongly disagree**

- “There is enough to deal with already. The thought of my phone telling me to go for a walk fills me with dread. Yet more to fail at.”

#### Future Interest

**Figure 1:**
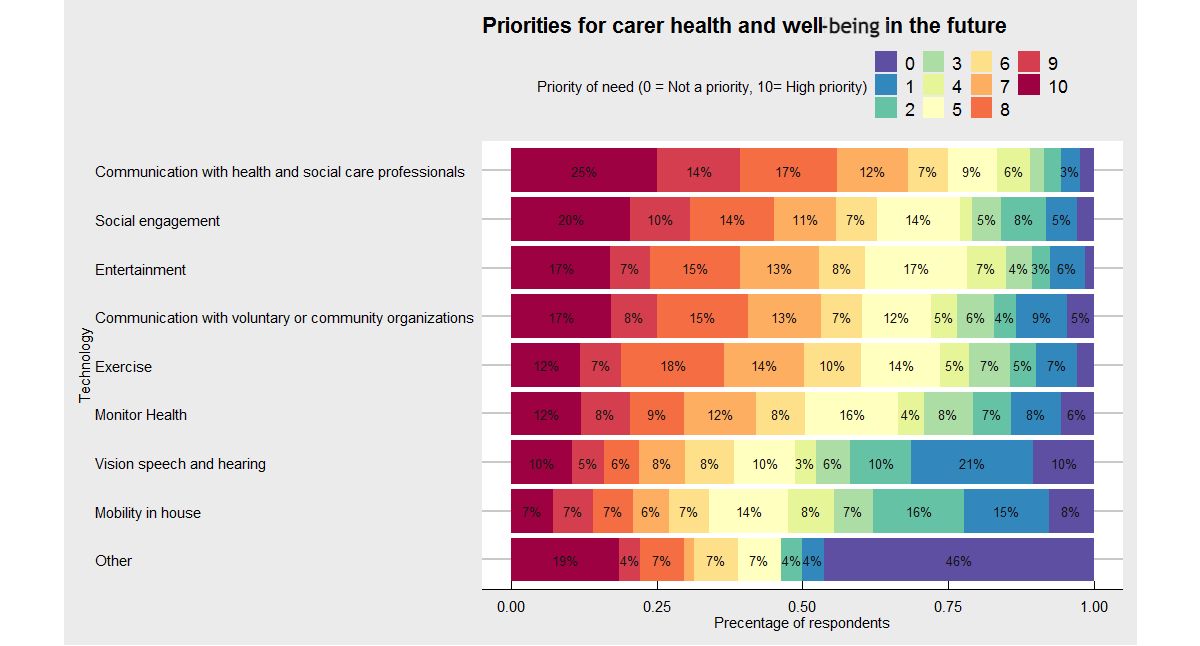
Summary figure of how caregivers would like to see future technologies support them with their own health and well-being in percentage. Color indicates priority of need where 0=not a priority (violet) and 10=highest priority (maroon).

When individuals were asked where they would like to see a focus on future technologies for caregiver health and well-being, we identified that there was a wide range of needs for future priorities (Figure 1). In parallel with the themes identified for current use, the most commonly reported future needs were around the themes of communication with health and social care professionals (62/247, 25.1%) of respondents rated this as the highest priority need). Other high-priority needs identified (ie, 10 out of a possible 10) for caregiver health and well-being were technologies focused on social engagement (51/245, 20.8%), entertainment (44/253, 17.4%), and communication with voluntary or community organizations (41/239, 17.2%). Some 54 respondents noted that not all of their needs were captured within the predefined eight categories. We explored these data and identified 24 diverse responses. Examples included, *accessing research and best practice recommendations* and *medication management*. Others were more interested in mental health aspects such as *rebuild my self-esteem* and *mental health monitoring advice and support.*

### Technology for Delivering Care

#### Current Interest

We gathered 238 responses on the agreement level regarding interest for caregivers to use technology within their caring role. We observed that 39.5% (94/238) strongly agreed, 33.6% (80/238) agreed, 21.8% (52/238) neither agreed nor disagreed, 3.4% (8/238) disagreed, and 1.5% (4/238) strongly disagreed. Through free text responses (Textbox 2), caregivers commonly noted several key benefits, including digital devices (eg, tablets, smartphones, or laptops) allow ease of access to information, checking in from distance (eg, Skype), supporting isolation, communication, entertainment (eg, Netflix), and help with simple reminders for care duties such as medications:

Caring comes down to people and we must get the focus back on to people, not technology!

Participant quote on using technology for caring role

Some caregivers reported knowledge of web-based learning and support modules. Concerns from caregivers included that reaching health and social care professionals remains difficult, and that the use of technology can be stressful for those cared for (eg, provoking anxiety). Technologies are also limited in their suitability for progressive conditions.

**Caregiver quotes for using technology in caring role. Caregivers were asked to rate their level of interest for using technology to help them with their caring role. Quotes are examples of further details given from participants grouped according to level of agreement.**

**Strongly agree**

- “In order for me to continue be able care at home I need technology. Simple as that.”

**Agree**

- “Living in a fairly isolated community and reliant on a car for appointments, shopping and visiting friends, technology such as video links, and FaceTime are helpful.”

**Neither agree nor disagree**

- “With dementia it is only useful in the early stages.”

**Disagree**

- “I care for someone with very complex mental health problems and technology would raise his already extreme anxiety I suspect.”

**Strongly disagree**

- “Caring comes down to people and we must get the focus back on to people, not technology!”

#### Type of Technology and Frequency of Use Within Caring Role

**Figure 2:**
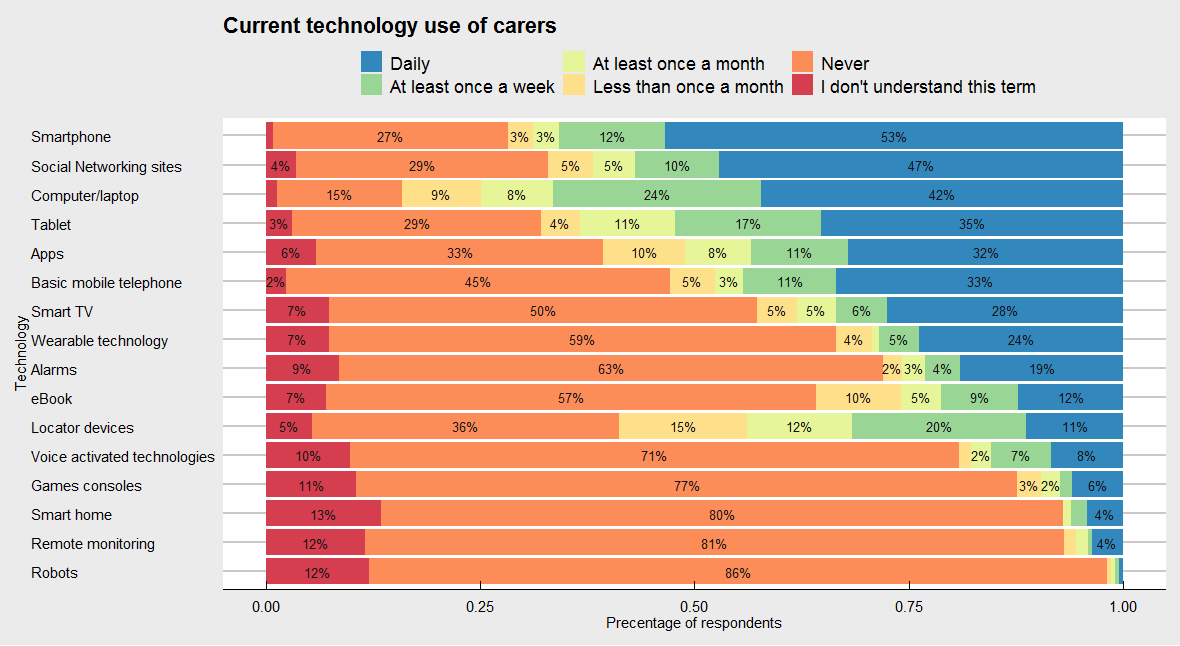
Current technology use of caregivers to conduct their caring role. The x-axis represents cumulative percentage use, whereas the y-axis represents the different types of technologies. Frequency of use was grouped into: (1) Daily, (2) At least once a week, (3) At least once a month, (4) Less than once a month, (5) Never and (5) I don’t understand this specific technology term. TV: television.

We asked caregivers about the type and frequency of technology they use for their caring role and they most commonly reported smartphones, computer or laptop use, and social networking sites with 65.8% (154/234), 66.5% (151/227), and 59.8% (128/214), respectively, reporting at least once weekly use (Figure 2). Many technologies have been used sporadically; for example, locator devices (eg, Google Maps and GPS) are used at least to some degree by 57.3% (130/227) of caregivers but are commonly used weekly or monthly. Regarding frequency of use, caregivers were twice as likely to have never used wearable technology as opposed to using it daily. The use of platforms specific for gaming was limited (eg, PlayStation or Xbox) and was used by 5.9% (13/218) of caregivers daily. Such use was not limited to younger ages but included involvement from caregivers aged 55 to 64 years. Perhaps tellingly, a lack of understanding of terminologies used in our survey was often associated with caregivers never using a specific technology to help them in their caring role (eg, robots, smart homes, and remote monitoring).

### Confidence and Support for Technology

We asked caregivers about their confidence levels when selecting the most appropriate technologies. Of the 238 responses, 25 (10.5%) strongly agreed, 82 (34.4%) agreed, 69 (28.9%) neither agreed nor disagreed, 51 (21.4%) disagreed, and 11 (4.6%) strongly disagreed that they were confident in selecting the most appropriate technologies for their caring role. A range of qualitative comments supported such statements (Textbox 3), particularly around barriers to adoption, such as that technology moves so fast, technology is often aimed at younger markets, and that information is lacking. Enablers for high confidence in selecting technology commonly involved caregivers who had a particular background in technology, a family member to hand with technology expertise, and the ability to search for solutions through computers or the internet:

No one has helped me. It was all down to Google and common sense.

Participant quote on confidence for using technology for caring role

I DO NOT want more technology in my caring role, there is more than enough and it is a failure

Participant quote on support for using technology for caring role

We also asked caregivers whether there was sufficient support and training for technology resources and services to help them in their caring role. Of the 236 responses, 6 (2.5%) strongly agreed, 33 (13.9%) agreed, 102 (43.2%) neither agreed nor disagreed, 67 (28.4%) disagreed, and 28 (11.9%) strongly disagreed. A range of qualitative comments supported these statements (Textbox 4), including financial restrictions, lack of visibility or existence of support, a need for self-sufficiency within the caring role, inability to accommodate all users (eg, older caregivers), and lack of overall support for caregivers with technology just being one component of this.

**Caregiver quotes regarding confidence for using technology in caring role. Caregivers rated their confidence about selecting the most appropriate technologies for their caring role. Quotes are extracts of further comments given from participants grouped according to level of agreement.**

**Strongly agree**

- “I like technology and try to find ways to adopt and adapt it for my use.”

**Agree**

- “I can find my way around most technologies.”

**Neither agree nor disagree**

- “Not always sure what will be most effective.”

**Disagree**

- “I just don’t know anything about what might be available.”

**Strongly disagree**

- “No one has helped me. It was all down to google and common sense.”

**Caregiver quotes regarding support and training for using technology in a caring role. Caregivers rated whether there was sufficient support and training for technology resources and services to help them in their caring role. Quotes are examples of further details given from participants grouped according to level of agreement.**

**Strongly agree**

- “I do not want more technology in my caring role, there is more than enough and it is a failure.”

**Agree**

- “I find I can access the information I need—I do wonder though, if it is as accessible to everyone?”

**Neither agree nor disagree**

- “Feel I have everything I need or can afford.”

**Disagree**

- “I have never been offered or had discussed any info on tech enabled care from anyone in Social Work or NHS.”

**Strongly disagree**

- “There is a general lack of support, let alone for tech stuff.”

### Health and Social Care Professional Interactions

**Figure 3:**
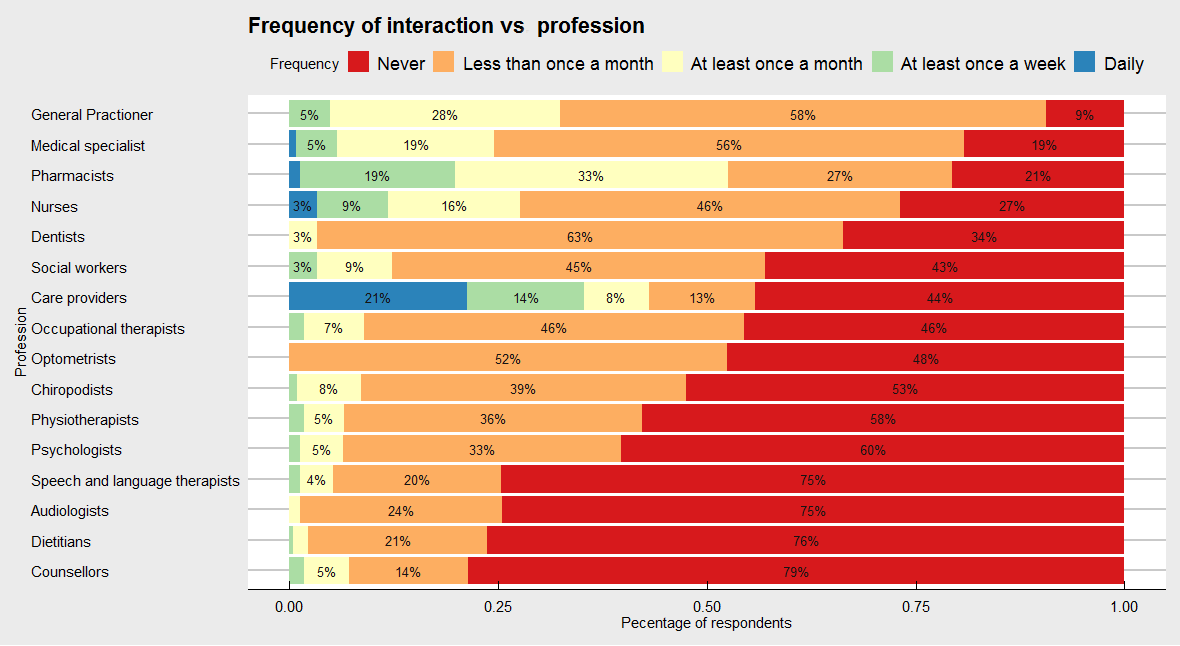
Frequency of health and social care professional interactions for caregivers. The x-axis represents cumulative percentage of interactions, whereas the y-axis represents the different health and social care professional groups Frequency of use was grouped into: (1) Daily, (2) At least once a week, (3) At least once a month, (4) Less than once a month, (5) Never.

We explored the types of health and social care professionals that caregivers interacted with as part of their caring role (Figure 3). The results revealed that a particularly common point of contact overall was the role of general practitioners, where 27.5% (73/265) reported interactions at least once a month and 4.9% (13/265) reported interactions at least once a week. Semiregular contact was made by 58.1% (154/265) of caregivers, whereas 9.4% (25/265) of respondents reported *never* interacting with this professional group. Pharmacists were a professional group that commonly interacted with caregivers, where 79.3% (192/242) of caregivers reported at least some interaction. More specifically, this included 1.2% (3/242) of caregivers who reported daily interactions, 18.6% (45/242) reported weekly interactions, and 32.6% (79/242) reported monthly interactions. Conversely, caregivers were less likely to interact with counselors and dietitians, with 78.7% (177/225) and 76.3% (171/224) of caregivers reported that they never interacted with these professional groups, respectively. The frequency of reach of some health and social care professionals was notably high within specific subgroups of caregivers. For example, although 55.7% (136/244) of respondents of caregivers interacted with care providers, it was common that these were very regular interactions (52/244, 21.3% daily and 34/244, 13.9% weekly).

### Future Interests

**Figure 4:**
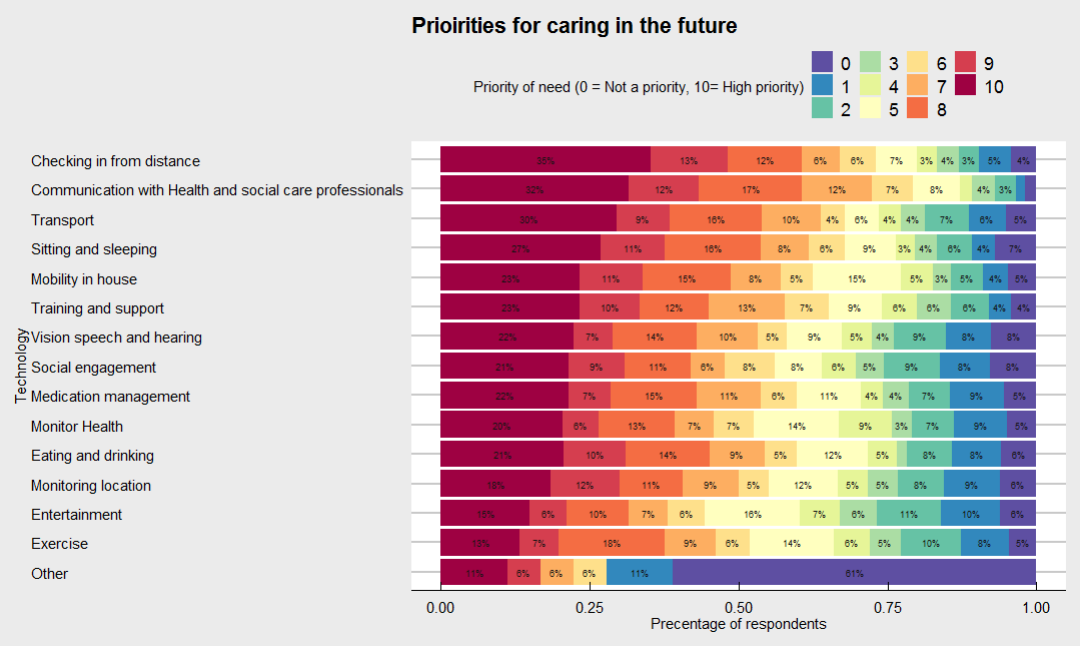
Priorities for future technologies to help caregivers to undertake their caring role. Color indicates priority of need where 0=not a priority (violet) and 10=highest priority (maroon).

When we asked caregivers about their priorities for caring, there was relatively little variance between many of the prespecified categories used (Figure 4). The most common priorities highlighted (ie, 10 of a possible 10) included: *checking in from distance* (65/185, 35.1%), *communication with health and social care professionals* (64/203, 31.5%), and *transport* (eg, help outside the house to move more easily and independently; 57/193, 29.5%). Interestingly, there was also a relatively strong need for activities of daily living (eg, sitting and sleeping; vision, speech, and hearing; and social engagement). Innovations regarding exercise and entertainment to help caregivers with their caring role were comparatively less desirable compared with other aspects, with 13.2% (26/197) and 14.9% (29/194) of caregivers stating this as a greater priority need.

**Figure 5:**
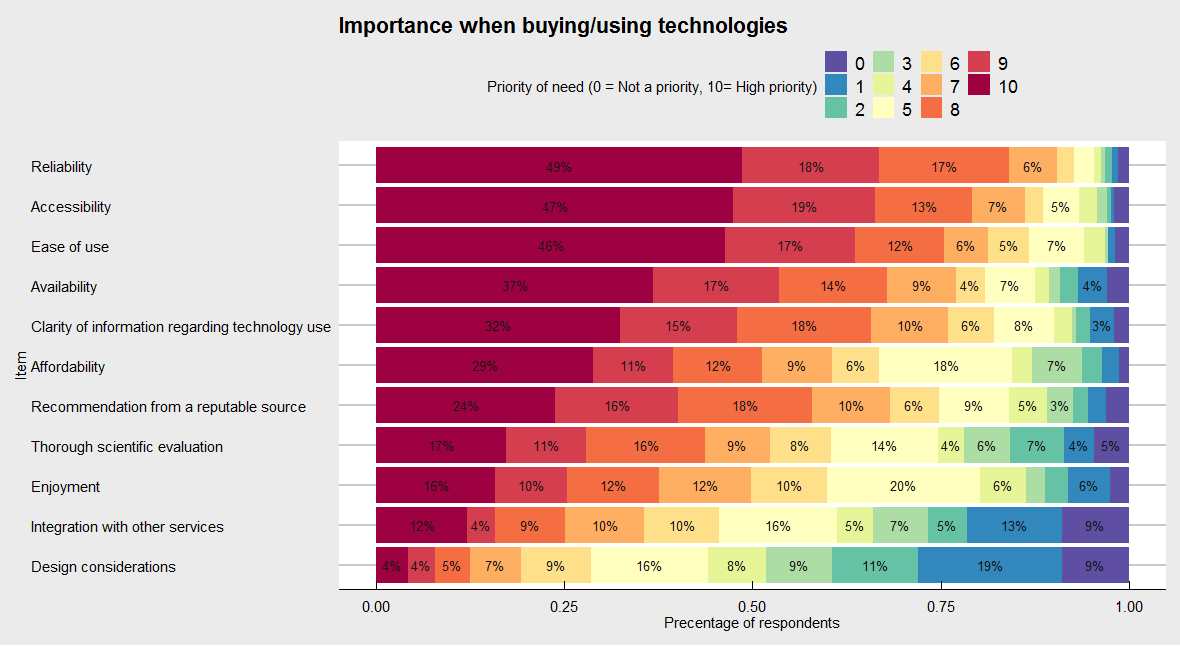
Priorities when deciding whether (or not) to buy future technologies. Color indicates priority of need where 0=not a priority (violet) and 10=highest priority (maroon).

We extended these questions to understand how caregivers make decisions about whether to purchase future technologies (Figure 5). Here, we identified that study participants most commonly allocated the highest desirability (ie, 10 of a possible 10) to reliability (107/220, 48.6%), ease of use (102/220, 46.4%), and accessibility (100/211, 47.4%). Comparatively, less important considerations were enjoyment of use (31/197, 15.7%), integration with other services (23/191, 12%), and design considerations (8/193, 4.1%).

## Discussion

### Principal Findings

We set out to improve collective knowledge on current and future technology use of caregivers, both for their own health and well-being, and the people they look after within the context of the United Kingdom. To our knowledge, our work is one of the largest surveys of its kind to focus specifically on the use of current and future digital health technologies to support the health and well-being of the UK caregivers and those cared for. Our sample was considerable in size, diversity, and level of detail recorded; caregivers varied in age, employment, gender, and conditions cared for. In agreement with cross-sectional evidence [3], our sample of caregivers was predominantly female; however, given sufficient sample size, it is of note that this finding may not always be paralleled across all age groups (eg, male caregivers are particularly common at the ages of ≥65 years [23]). The findings suggest that technologies play a diverse role in informal care settings where most, but not all, caregivers are largely positive about the potential. Such positivity is not without reservation, as technology can also be a negative force. Ongoing support is clearly lacking, and real-world value and implementation is limited as caregivers face challenges across cost, sustainability, availability, and reliability.

### Limitations

Our study had several limitations. First, given that caregivers are often hidden within the society, selection biases remain challenging to avoid. For example, our self-reported ethnicity data reflect the UK national statistics, where 94% of UK caregivers identify as White [24]. Moreover, as critical questions remain regarding the extent of the additional challenges that ethnic minorities face, it is pertinent that both research and policy strategies continue to reach these groups. Most of our survey respondents were highly educated; however, this may not be representative of the general population. Work conducted by the New Policy Institute in 2014 (based on the Office of National Statistics Family Resources Survey) found that 70% of working-age people caring for 20 hours a week or more did not have qualifications above the General Certificate of Secondary Education level [24]. Our methods of recruitment (using existing social networks, such as Twitter) may also have encouraged specific conditions (eg, dementia and cancer) or populations (eg, greater number of years of formal education) to respond. Our postal replies were few, and we did not explore how individuals found our initial web link to the survey in detail. Nevertheless, our work still represented over 20 different conditions, and 14% of those cared for were under the age of 16. Understanding caregiver needs in more rare conditions remains vital [25]; however, it falls out of the scope of this work. Although the development of a survey promoted on the web has helped us reach many people in a relatively short time frame, those averse to digital health technologies may be underrepresented. There are also inherent biases associated with convenience sampling; our participants were predominately based in Scotland, reflecting our local links and networks. Very few of our participants were new to caregiving. Although the extensive experience of our respondents is advantageous for gaining long-term insights, further work is needed to understand how challenges differ within those who are new to caregiving, particularly with respect to information seeking. Finally, to ensure anonymity of responses, we are not able to explore geographic, socioeconomic, or deprivation indices in further detail: important avenues for future work.

### Interpretation and Future Directions

There are several findings from this work that are pertinent to understanding caregiver demographics. Here, we build on technology use for caregivers by exploring in detail what makes caregivers receptive to technology across confidence, support, exploring health professionals involved, and the drive to look after their own health and well-being both now and in the future [26,27]. Most caregivers who responded to our survey regularly used smartphones, social networking sites, and computers or laptops to support their caring activities at least once a week (with many using such devices daily). This work adds to the current evidence that caregivers are not simply an *extension* of the health and social care service, but a diverse group given little attention and support [16]. Our results have demonstrated that caregivers do not just crave but also need much stronger and more meaningful links to our health and care professionals, which hold particular weight given the context of COVID-19 and risk of future pandemics. Technology could easily support such links, but the risk of rejection from professional health staff and caregivers could severely impact implementation. Careful, caregiver-led solution design is nonnegotiable if we are to support those most in need, including those who are isolated or have less experience in digital and health literacy. Particularly useful points of contact for this could include signposting and some limited support from: general practitioners, those working within professional caring roles, or a pharmacy setting.

This work highlights lessons in that regularity of use may not always represent perceived usefulness, for example, many caregivers make use of locator devices (eg, Google Maps and GPS) and laptops weekly or monthly (opposed to daily). It could be particularly fruitful to understand such relationships in more depth within future work and why many apps and technology solutions become left *behind.* Both digital divide and health literacy levels are important considerations. Despite sharing brief descriptors, concepts such as *Internet of Things* devices or *robots* can be alien to respondents and, accordingly, few participants reported use. Further work is now required to (1) ensure that caregivers are provided with knowledge and awareness of what technology is available; (2) achieve sustainable models of support; and (3) identify how research and policy can extend the utility of both new and existing technologies, including isolated or poorly represented groups.

Throughout the work conducted, there were several notable concerns raised about current and future technologies (eg, costs and timeliness of solutions), which aligns with other literatures [28]. Both our own findings and others [29-31] have highlighted the high degree of isolation that caregivers face. Successful innovation and technologies are strategies that can tackle this problem, including connecting caregivers to supportive environments, such as family, friends, and health and social care professionals. Furthermore, our work parallels both policy and research findings elsewhere (eg, in dementia technology charters, web-based resources, and the UK government policy documents) that technology should not replace human contact [32-34]. Given that a shrinking UK health care workforce appears all but inevitable [35], innovative solutions (both technology-based and otherwise) are required. This work highlights that caregivers need to be involved from concept initiation all the way through to the postevaluation stages.

Our work also informs state-level actors and health and social care providers. Caregivers demonstrated confidence in choosing technologies for their needs, but the need for support to use technologies was highlighted throughout. However, the delivery of this objective is complex. There are key questions regarding *who* should provide this support in the longer term. Although the potential for care providers and pharmacists to deliver technology supports is clear (ie, professionals who regularly interact with caregivers), careful consideration of workload, resources and training, and overall interest and acceptance is required. In addition, it is somewhat concerning that so few individuals are able to access the vital (and often preventive) support delivered by other professionals, such as counselors and nutritionists. The absence of access may not necessarily be associated with the absence of need. Perhaps indicative of the high need for technology solutions is that caregivers ranked *thorough scientific evaluation* relatively low regarding priority. Taken together, there is an urgent need to protect caregivers from purchasing unproven or unsafe technologies to bridge gaps in care, as highlighted in recent dementia reviews [36].

Finally, the collective message from caregivers is that having a wide array of unsupported gadgets (new or existing) cannot address core needs in day-to-day caregiving. Well-established technologies are still not reaching caregivers in a satisfactory form (eg, checking in for distance and communication tools). Caregivers frequently face health and well-being challenges alone, highlighted by a need for *communication with health and social care professionals* and are urgently looking for solutions regardless of the quality of science or how personalized technologies can be made. Continued co-design and consultation is required to improve current and future systems and technologies in a transparent manner, particularly given the significant reform and change that is well underway [37].

### Conclusions

Digital technologies appear to be largely acceptable for caregivers. As we look to the future, this work suggests that caregivers are calling for solutions that augment the human touch, connecting caregivers to those cared for (including at distance), friends and family, and health and social care professionals. Quality is key: unsupported and unreliable technologies remain problematic (and may not enhance safety or well-being), where finding and using technologies is often compounded by time pressures. Technological developments remain fragmented, and it is critical that new horizons collectively deliver on empowering caregivers with skillsets, knowledge, and tools to help their day-to-day role. Moreover, this work reiterates the need for all stakeholders, including academics, policy makers, and practitioners, to recognize the invaluable role that caregivers play in communities and to ensure that this group become equal coarchitects of the emerging digital health agenda.

## Appendix

Multimedia Appendix 1 Survey example distributed to study participants (paper format).

Multimedia Appendix 2 The number of different health conditions cared for across the sample.

## References

[1] (2015). Valuing carers 2015. Carers UK.

[2] (2017). Unpaid carers provide social care worth £57 billion. Office for National Statistics.

[3] Calvó-Perxas L, Vilalta-Franch J, Litwin H, Turró-Garriga O, Mira P, Garre-Olmo J (2018). What seems to matter in public policy and the health of informal caregivers? A cross-sectional study in 12 European countries. PLoS One.

[4] (2016). Missing Out, the identification challenge. Carers UK.

[5] (2019). Juggling work and unpaid care: a growing issue. Carers UK.

[6] de Labra C, Millán-Calenti JC, Buján A, Núñez-Naveira L, Jensen AM, Peersen MC, Mojs E, Samborski W, Maseda A (2015). Predictors of caregiving satisfaction in informal caregivers of people with dementia. Arch Gerontol Geriatr.

[7] Douglas SL, Mazanec P, Lipson A, Leuchtag M (2016). Distance caregiving a family member with cancer: a review of the literature on distance caregiving and recommendations for future research. World J Clin Oncol.

[8] Roth DL, Fredman L, Haley WE (2015). Informal caregiving and its impact on health: a reappraisal from population-based studies. Gerontologist.

[9] Capistrant BD (2016). Caregiving for older adults and the caregivers’ health: an epidemiologic review. Curr Epidemiol Rep.

[10] Irfan B, Irfan O, Ansari A, Qidwai W, Nanji K (2017). Impact of caregiving on various aspects of the lives of caregivers. Cureus.

[11] (2016). Families caring for an aging America. National Academies of Sciences, Engineering, and Medicine.

[12] Ringer TJ, Wong-Pack M, Miller P, Patterson C, Marr S, Misiaszek B, Woo T, Sztramko R, Vastis PG, Papaioannou A (2018). Understanding the educational and support needs of informal care-givers of people with dementia attending an outpatient geriatric assessment clinic. Ageing Soc.

[13] (2015). The health and wellbeing of unpaid carers: where can digital skills and community support add value?. BASW.

[14] 2018 Global health care outlook. Deloitte.

[15] Topol EJ (2019). High-performance medicine: the convergence of human and artificial intelligence. Nat Med.

[16] Lindeman D, Kim K, Gladstone C, Apesoa-Varano E (2020). Technology and caregiving: emerging interventions and directions for research. Gerontologist.

[17] Heynsbergh N, Heckel L, Botti M, Livingston PM (2018). Feasibility, useability and acceptability of technology-based interventions for informal cancer carers: a systematic review. BMC Cancer.

[18] Tieu L, Sarkar U, Schillinger D, Ralston JD, Ratanawongsa N, Pasick R, Lyles CR (2015). Barriers and facilitators to online portal use among patients and caregivers in a safety net health care system: a qualitative study. J Med Internet Res.

[19] (2016). AARP.

[20] ALLIANCE.

[21] Carers UK.

[22] Braun V, Clarke V (2006). Using thematic analysis in psychology. Qual Res Psychol.

[23] (2017). Unpaid carers provide social care worth £57 billion. Office of National Statistics Website.

[24] Research briefing. UK Parliment.

[25] Mooney J, Graham K, Watts R (2019). Impact of caring for someone with a rare rheumatic condition, views from patients and informal carers-the need for cat-like vigilance. Rheumatol Adv Pract.

[26] Egan KJ, Pot AM (2016). Encouraging innovation for assistive health technologies in dementia: barriers, enablers and next steps to be taken. J Am Med Dir Assoc.

[27] Madara Marasinghe K (2016). Assistive technologies in reducing caregiver burden among informal caregivers of older adults: a systematic review. Disabil Rehabil Assist Technol.

[28] Mortenson WB, Pysklywec A, Fuhrer MJ, Jutai JW, Plante M, Demers L (2018). Caregivers' experiences with the selection and use of assistive technology. Disabil Rehabil Assist Technol.

[29] Vasileiou K, Barnett J, Barreto M, Vines J, Atkinson M, Lawson S, Wilson M (2017). Experiences of loneliness associated with being an informal caregiver: a qualitative investigation. Front Psychol.

[30] Hussain R, Wark S, Ryan P (2018). Caregiving, employment and social isolation: challenges for rural carers in Australia. Int J Environ Res Public Health.

[31] Greenwood N, Pound C, Smith R, Brearley S (2019). Experiences and support needs of older carers: a focus group study of perceptions from the voluntary and statutory sectors. Maturitas.

[32] Vaughan C, Trail TE, Mahmud A, Dellva S, Tanielian T, Friedman E (2018). Informal caregivers' experiences and perceptions of a web-based peer support network: mixed-methods study. J Med Internet Res.

[33] (2016). Technology charter: for people living with dementia in Scotland. Dementia Partnership.

[34] Robotics in social care. New Electronics.

[35] (2019). A critical moment: NHS staffing trends, retention and attrition. The Health Foundation.

[36] Livingston G, Sommerlad A, Orgeta V, Costafreda SG, Huntley J, Ames D, Ballard C, Banerjee S, Burns A, Cohen-Mansfield J, Cooper C, Fox N, Gitlin LN, Howard R, Kales HC, Larson EB, Ritchie K, Rockwood K, Sampson EL, Samus Q, Schneider LS, Selbæk G, Teri L, Mukadam N (2017). Dementia prevention, intervention, and care. Lancet.

[37] A digital NHS? An introduction to the digital agenda and plans for implementation. The Kings Fund.

