# Supplementary material for: Understanding Current Needs and Future Expectations of Informal Caregivers for Technology to Support Health and Well-being: National Survey Study
Source: JMIR Aging. 2022 Jan 27;5(1):e15413. doi: 10.2196/15413 (PMC8832269; doi:10.2196/15413)
Supplement: Multimedia Appendix 1 [file aging_v5i1e15413_app1.docx]

**Multimedia Appendix 1.** Survey example distributed to study participants (paper format).

**Part 1: YOU AS A CARER**

*This section is focused on building up an understanding of you.*

We understand that all carers are different: some of you may not view yourself as a carer, but are still looking after another person at home or outside of clinical settings. Throughout this questionnaire, where we use the term 'carer' we mean a person (usually a family member friend or relative) who is looking after an ill, older or disabled family member, friend or partner. As all carers are different, this section is designed to help us understand some basic information about YOU AS A CARER.

How old are you in years? Please select one.

- 18 to 24
- 25 to 34
- 35 to 44
- 45 to 54
- 55 to 64
- 65 to 74
- 75 to 84
- 85+
- Prefer not to say

What is your gender? Please select one.

- Male
- Female
- Other
- Prefer not to say

What is your highest level of education? Please select one.

- Degree or equivalent
- Higher education
- School qualifications
- Other qualifications
- No qualifications
- Don't know

]

What is your ethnicity? Please select one.

- White
- Mixed/multiple ethnic groups
- Asian/Asian British
- Black/African/Caribbean/Black British
- Other ethnic group
- Prefer not to say

How long have you been a carer? *Please state years and/or months with your answer*

_______________________________________________________________________________

Are you still able to work as you care?

- Yes, part time
- Yes, full time
- Caregiving has caused me to give up work
- I do not currently work and did not have to give up work due to caregiving

Which part of the UK do you live in?

- England
- Northern Ireland
- Scotland
- Wales

**Part 2: YOU AS A CARER**

*This section is focused on building up an understanding of you as a carer and how technology could assist you.*

When looking at your caregiving role as a whole, it might be that you don't tend to think about your own health and wellbeing as a priority. But it is very common that caregivers face their own mental and physical health challenges, so we are interested to understand this in more detail. Please note that the questions in this section are about **YOU AS A CARER**and how technology can support you to stay as well as possible.

**Definition of technologies in healthcare**

Technology devices are used across a variety of health and wellbeing interests. This includes electronic devices such as: smartphones, iPads , mobile 'apps' and wearable technologies. For a visual example please see the picture below.

Examples of technology that could help you look after your own health and wellbeing can include:

- Mobile phone applications that help with motivation or measurement of exercise (e.g. "couch to 5k")

- Wearable technologies (e.g. apple watch, Fitbit)

- Medication management (e.g. electronic pill box)

- Entertainment use (e.g. audiobooks, Netflix, an Alexa based quiz ) 

If you would like further information on this topic please visit: 
  
<https://www.carersuk.org/help-and-advice/technology-and-equipment>


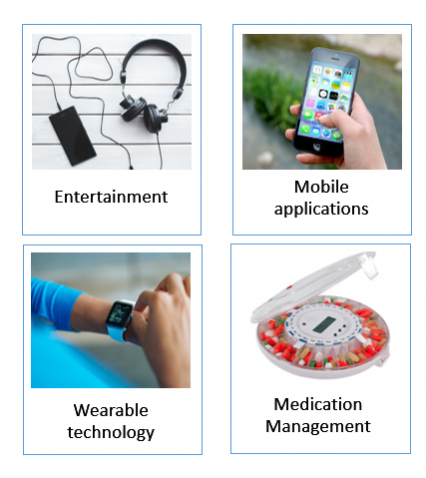


I am interested in technology to help me with my **own health and wellbeing**

- Strongly agree
- Agree
- Neither agree nor disagree
- Disagree
- Strongly disagree

Please describe your views on using technology for your **own health and wellbeing**

_______________________________________________________________________________

_______________________________________________________________________________

_______________________________________________________________________________

_______________________________________________________________________________

_______________________________________________________________________________

**To help me look after my own health and wellbeing in the future,**I would like to see future technologies support me with the following

(Please circle the priority of need using scale of 1 to 10 below)

| **Item** | **Low priority** | | |  |  | **Medium priority** | |  |  | **High priority** | |
| --- | --- | --- | --- | --- | --- | --- | --- | --- | --- | --- | --- |
| Entertainment and/or relaxation (e.g. delivery of audio-books, quizzes, learning, mindfulness videos, laughing yoga apps) | 0 | 1 | 2 | 3 | 4 | 5 | 6 | 7 | 8 | 9 | 10 |
| Exercise (e.g. exercises that I could do at home) | 0 | 1 | 2 | 3 | 4 | 5 | 6 | 7 | 8 | 9 | 10 |
| Mobility in the house (e.g. automatic lights) | 0 | 1 | 2 | 3 | 4 | 5 | 6 | 7 | 8 | 9 | 10 |
| Monitoring health (e.g. wearable heart rate monitor) | 0 | 1 | 2 | 3 | 4 | 5 | 6 | 7 | 8 | 9 | 10 |
| Social engagement (e.g. allow me as a carer to connect with others over the internet) | 0 | 1 | 2 | 3 | 4 | 5 | 6 | 7 | 8 | 9 | 10 |
| Communication with heath and social care professionals about my own health (e.g. communicating the physical and mental impacts of caregiving) | 0 | 1 | 2 | 3 | 4 | 5 | 6 | 7 | 8 | 9 | 10 |
| Vision, speech and hearing (e.g. improved technologies to help me with my sight, hearing or voice) | 0 | 1 | 2 | 3 | 4 | 5 | 6 | 7 | 8 | 9 | 10 |
| Communication with voluntary or community organizations/groups about my own health (e.g. communicating the physical and mental impacts of caregiving) | 0 | 1 | 2 | 3 | 4 | 5 | 6 | 7 | 8 | 9 | 10 |
| Other* | 0 | 1 | 2 | 3 | 4 | 5 | 6 | 7 | 8 | 9 | 10 |

***If you selected "Other" for the question above, please explain:**

_______________________________________________________________________________

_______________________________________________________________________________

_______________________________________________________________________________

_______________________________________________________________________________

_______________________________________________________________________________

**Part 3.1: The person (s) that you care for**

*This section is focused on building up an understanding of THE PERSON(S) THAT YOU CARE FOR*

We understand that people who are cared for are all different. This section is designed to help us understand some basic information about the person that you care for. 
 
On this page we first ask about the **number of people that you care for.**
 

**If you care for more than one person, you will be asked to add some basic details about EACH person you are caring for (age, condition, etc.). Please fill in the first person that you care for in the main questionnaire and you can find additional individuals cared for in Annex 1 at the end of this booklet.**

**If you care for more than 4 people, please select the 4 main people you care for.**

How many people do you care for?

- 1
- 2
- 3
- 4 or more

**PERSON 1 that you care for**

How old is the person that you care for in years? Please select one.

- 0 to 15
- 16 to 24
- 25 to 34
- 35 to 44
- 45 to 54
- 55 to 64
- 65 to 74
- 75 to 84
- 85+
- Prefer not to say

What is the gender of the person that you care for? Please select one.

- Male
- Female
- Other
- Prefer not to say

What is the ethnicity of the person that you care for? Please select one.

- White
- Mixed/multiple ethnic groups
- Asian/Asian British
- Black/African/Caribbean/Black British
- Other ethnic group
- Prefer not to say

What condition(s) does the person that you care for have? Please tick all that apply.

- An autism spectrum disorder
- Asthma
- Arthritis
- Alcohol or substance misuse
- Coronary heart disease/heart failure
- Chronic Obstructive Pulmonary Disease (COPD)
- Cancer
- Dementia
- Diabetes
- A learning disability
- A mental health condition
- Needs that arise from being older (e.g. support with mobility)
- Another neurological condition (e.g Parkinson's, Motor Neurone Disease or MS)
- A physical disability
- They require palliative or end of life care
- A sensory impairment
- Stroke
- Other long term health condition
- Other (please specify)*
- A combination of conditions or disabilities
- Prefer not to say

*If you selected "other" or you would like to share further details of the health conditions the person that you care for has, please write them here

_______________________________________________________________________________

_______________________________________________________________________________

_______________________________________________________________________________

_______________________________________________________________________________

_______________________________________________________________________________

_______________________________________________________________________________

The person that I care for has problems with the following

- Blind or sight loss
- Deaf or hearing loss
- Speech impairment
- Precise hand movements
- Other
- Prefer not to say

If you selected "other" or you would like to share further details of the health problems the person that you care for has, please write them here

_______________________________________________________________________________

_______________________________________________________________________________

_______________________________________________________________________________

_______________________________________________________________________________

_______________________________________________________________________________

**If you care for more than one person, please now fill out Annex 1, otherwise please continue to Part 3.2**

**Part 3.2: The person (s) that you care for**

Through my caring role, I interact with the following health and social care professionals:

| **Professional** | **Daily** | **At least once a week** | **At least once a month** | **Less than once a month** | **Never** |
| --- | --- | --- | --- | --- | --- |
| General Practitioner | ⃝ | ⃝ | ⃝ | ⃝ | ⃝ |
| Medical specialist | ⃝ | ⃝ | ⃝ | ⃝ | ⃝ |
| Nurses | ⃝ | ⃝ | ⃝ | ⃝ | ⃝ |
| Occupational therapists | ⃝ | ⃝ | ⃝ | ⃝ | ⃝ |
| Psychologists | ⃝ | ⃝ | ⃝ | ⃝ | ⃝ |
| Audiologists | ⃝ | ⃝ | ⃝ | ⃝ | ⃝ |
| Optometrists | ⃝ | ⃝ | ⃝ | ⃝ | ⃝ |
| Dentists | ⃝ | ⃝ | ⃝ | ⃝ | ⃝ |
| Physiotherapists | ⃝ | ⃝ | ⃝ | ⃝ | ⃝ |
| Chiropodists | ⃝ | ⃝ | ⃝ | ⃝ | ⃝ |
| Speech and language therapists | ⃝ | ⃝ | ⃝ | ⃝ | ⃝ |
| Counsellors | ⃝ | ⃝ | ⃝ | ⃝ | ⃝ |
| Dietitians | ⃝ | ⃝ | ⃝ | ⃝ | ⃝ |
| Pharmacists | ⃝ | ⃝ | ⃝ | ⃝ | ⃝ |
| Social workers | ⃝ | ⃝ | ⃝ | ⃝ | ⃝ |
| Care providers | ⃝ | ⃝ | ⃝ | ⃝ | ⃝ |
| Other* | ⃝ | ⃝ | ⃝ | ⃝ | ⃝ |

*If you selected "Other" please describe in further detail here:

_______________________________________________________________________________

_______________________________________________________________________________

**Part 4: The person(s) that you care for continued**

*This section is focused on building up an understanding of the use of technology for****the person(s) that you are caring for both now and in the future***

Technology to help support you care for another person.
 
As a quick reminder, there are lots of different technologies that you can use to help the person that you care for. This includes electronic devices such as: smartphones, iPads , mobile 'apps' and wearable technologies. For a visual example please see the picture below. Some of these technologies might be the same that you would use to monitor and improve your own health, whereas others might be different. **For all the questions in this section we are interested in both technologies that you might use with the person you are caring for (i.e a device you use together), and technologies that the person you are caring for might use on their own (i.e. a device the person you care for can use alone).**

  Examples of technology use can include:
 
- Monitoring in your home for independent living (e.g. using movement sensors, or electricity meters)
 -Remote monitoring and alerts for times where you are not with the person you care for (e.g. telehealth)
- Wearable technology (e.g. heart rate/breathing)
- Medication management (e.g. electronic pill box)
- Entertainment (e.g. audiobooks, games) 
 
If you would like further information on this topic please visit:
<https://www.carersuk.org/help-and-advice/technology-and-equipment>


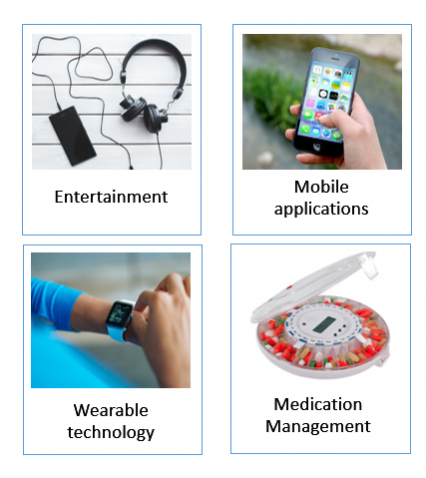


I am interested in technology to help me with **my caring role**

- Strongly agree
- Agree
- Neither agree nor disagree
- Disagree
- Strongly disagree

Please describe your views on using technology for **your caring role** here

_______________________________________________________________________________

_______________________________________________________________________________

_______________________________________________________________________________

_______________________________________________________________________________

**Current technology use**

In your caring role, how often do you use the following technologies? Please complete the following for each technology device and if you don't use something in your caring role, use the 'never' option.

|  | Daily | At least once a week | At least once a month | Less than once a month | Never | I do not understand this specific technology term |
| --- | --- | --- | --- | --- | --- | --- |
| Smartphone (e.g. a phone that you can download apps to) | ⃝ | ⃝ | ⃝ | ⃝ | ⃝ | ⃝ |
| Basic mobile telephone | ⃝ | ⃝ | ⃝ | ⃝ | ⃝ | ⃝ |
| Computer/laptop | ⃝ | ⃝ | ⃝ | ⃝ | ⃝ | ⃝ |
| Tablet (e.g. iPad) | ⃝ | ⃝ | ⃝ | ⃝ | ⃝ | ⃝ |
| eBook (e.g. Kindle) | ⃝ | ⃝ | ⃝ | ⃝ | ⃝ | ⃝ |
| ‘Apps’ (e.g. downloaded from Google Play/the App Store) | ⃝ | ⃝ | ⃝ | ⃝ | ⃝ | ⃝ |
| Social networking sites (e.g. Facebook, Twitter) | ⃝ | ⃝ | ⃝ | ⃝ | ⃝ | ⃝ |
| Games console (e.g. Playstation, Xbox) | ⃝ | ⃝ | ⃝ | ⃝ | ⃝ | ⃝ |
| Locator devices (e.g. Google Maps, GPS) | ⃝ | ⃝ | ⃝ | ⃝ | ⃝ | ⃝ |
| Smart home (e.g. two or more connected 'smart' devices such as using Hive with heating) | ⃝ | ⃝ | ⃝ | ⃝ | ⃝ | ⃝ |
| Smart TV (e.g. TV with 'smart' connectivity and internet access) | ⃝ | ⃝ | ⃝ | ⃝ | ⃝ | ⃝ |
| Remote monitoring (home monitoring of the physical health and/or daily patterns of the person cared for) | ⃝ | ⃝ | ⃝ | ⃝ | ⃝ | ⃝ |
| Wearable technology (e.g. fitbit, pendant alarm) | ⃝ | ⃝ | ⃝ | ⃝ | ⃝ | ⃝ |
| Voice activated technologies (e.g. Siri, Alexa, Google Home) | ⃝ | ⃝ | ⃝ | ⃝ | ⃝ | ⃝ |
| Robots (e.g. hoover. robotic seal) | ⃝ | ⃝ | ⃝ | ⃝ | ⃝ | ⃝ |
| Alarms (e.g. safety alarm, falls alarm) | ⃝ | ⃝ | ⃝ | ⃝ | ⃝ | ⃝ |

If the technology device that you use in **your caring role** is not listed above, please add it below:

_______________________________________________________________________________

_______________________________________________________________________________

_______________________________________________________________________________

_______________________________________________________________________________

There is sufficient support and training for technology resources and services to help me in **my caring role**

- Strongly agree
- Agree
- Neither agree nor disagree
- Disagree
- Strongly disagree

Please explain:

_______________________________________________________________________________

_______________________________________________________________________________

_______________________________________________________________________________

I feel confident to select the most appropriate technologies to assist me **in my caring role**

- Strongly agree
- Agree
- Neither agree nor disagree
- Disagree
- Strongly disagree

Please explain:

_______________________________________________________________________________

_______________________________________________________________________________

_______________________________________________________________________________

Use the scale below to rate how important each of the following items is when deciding whether to buy/use a technology **in my caring role.**

| Item | Low priority | | |  |  | Medium priority | |  |  | High priority | |
| --- | --- | --- | --- | --- | --- | --- | --- | --- | --- | --- | --- |
| Affordability | 0 | 1 | 2 | 3 | 4 | 5 | 6 | 7 | 8 | 9 | 10 |
| Reliability (e.g. does the device always work as intended) | 0 | 1 | 2 | 3 | 4 | 5 | 6 | 7 | 8 | 9 | 10 |
| Integration with other services (e.g. Alexa working in partnership with community groups or social services) | 0 | 1 | 2 | 3 | 4 | 5 | 6 | 7 | 8 | 9 | 10 |
| Design considerations (e.g. colour, aesthetics. size) | 0 | 1 | 2 | 3 | 4 | 5 | 6 | 7 | 8 | 9 | 10 |
| Clarity of information regarding the use of the technology | 0 | 1 | 2 | 3 | 4 | 5 | 6 | 7 | 8 | 9 | 10 |
| Recommendation from a reputable source | 0 | 1 | 2 | 3 | 4 | 5 | 6 | 7 | 8 | 9 | 10 |
| Thorough scientific evaluation | 0 | 1 | 2 | 3 | 4 | 5 | 6 | 7 | 8 | 9 | 10 |
| Availability (e.g. is the device available in my area) | 0 | 1 | 2 | 3 | 4 | 5 | 6 | 7 | 8 | 9 | 10 |
| Accessibility (e.g. is the device appropriate for my needs) | 0 | 1 | 2 | 3 | 4 | 5 | 6 | 7 | 8 | 9 | 10 |
| Enjoyment | 0 | 1 | 2 | 3 | 4 | 5 | 6 | 7 | 8 | 9 | 10 |
| Ease of use | 0 | 1 | 2 | 3 | 4 | 5 | 6 | 7 | 8 | 9 | 10 |

**Future technology use**

**To help me in my caring role in the future,** I need technologies to help me with the following (Please rate the priority of need using sliding scale)

*If you selected "Other" please describe in further detail here:

__________________________________________________________________________________

__________________________________________________________________________________

__________________________________________________________________________________

| **Item** | **Low priority** | | |  | **Medium priority** | | |  | **High**  **priority** | | |
| --- | --- | --- | --- | --- | --- | --- | --- | --- | --- | --- | --- |
| Entertainment (e.g. delivery of audio-books, quizzes, learning) | 0 | 1 | 2 | 3 | 4 | 5 | 6 | 7 | 8 | 9 | 10 |
| Exercise (e.g. exercises that the person I care for could do at home) | 0 | 1 | 2 | 3 | 4 | 5 | 6 | 7 | 8 | 9 | 10 |
| Medication management (e.g. help to administer daily prescriptions) | 0 | 1 | 2 | 3 | 4 | 5 | 6 | 7 | 8 | 9 | 10 |
| Eating and drinking (E.g. technology that could make it easier to help the person I care for be more independent at mealtimes) | 0 | 1 | 2 | 3 | 4 | 5 | 6 | 7 | 8 | 9 | 10 |
| Mobility in the house (e.g. making the house safer to move around, such as automatic lights) | 0 | 1 | 2 | 3 | 4 | 5 | 6 | 7 | 8 | 9 | 10 |
| Monitoring health (e.g. wearable heart rate monitor) | 0 | 1 | 2 | 3 | 4 | 5 | 6 | 7 | 8 | 9 | 10 |
| Social engagement (e.g. allow the person I care for to connect with others over the internet) | 0 | 1 | 2 | 3 | 4 | 5 | 6 | 7 | 8 | 9 | 10 |
| Sitting and sleeping (e.g. help improve the duration or quality of sleep for the person I care for) | 0 | 1 | 2 | 3 | 4 | 5 | 6 | 7 | 8 | 9 | 10 |
| Transport (e.g. help the person I care for move around outside the house more easily/independently) | 0 | 1 | 2 | 3 | 4 | 5 | 6 | 7 | 8 | 9 | 10 |
| Communication with heath and social care professionals (e.g. reporting aspects of health for the person I am caring for directly with clinicians) | 0 | 1 | 2 | 3 | 4 | 5 | 6 | 7 | 8 | 9 | 10 |
| Vision, speech and hearing (e.g. improved technologies to help with basic needs for the person that I am caring for) | 0 | 1 | 2 | 3 | 4 | 5 | 6 | 7 | 8 | 9 | 10 |
| Training and support (e.g. a person or organization that can help you use the device on a regular basis) | 0 | 1 | 2 | 3 | 4 | 5 | 6 | 7 | 8 | 9 | 10 |
| Monitoring mobility/activity such as tracking location (e.g. GPS), energy use and fridge sensors | 0 | 1 | 2 | 3 | 4 | 5 | 6 | 7 | 8 | 9 | 10 |
| Monitoring/assistive device that helps me care or check in with the person from a distance to give me peace of mind | 0 | 1 | 2 | 3 | 4 | 5 | 6 | 7 | 8 | 9 | 10 |
| Other* | 0 | 1 | 2 | 3 | 4 | 5 | 6 | 7 | 8 | 9 | 10 |

**Annex 1: The person (s) that you care for (to complete where caring for more than one person)**

**PERSON 2 that you care for**

How old is the person that you care for in years? Please select one.

- 0 to 15
- 16 to 24
- 25 to 34
- 35 to 44
- 45 to 54
- 55 to 64
- 65 to 74
- 75 to 84
- 85+
- Prefer not to say

What is the gender of the person that you care for? Please select one.

- Male
- Female
- Other
- Prefer not to say

What is the ethnicity of the person that you care for? Please select one.

- White
- Mixed/multiple ethnic groups
- Asian/Asian British
- Black/African/Caribbean/Black British
- Other ethnic group
- Prefer not to say

What condition(s) does the person that you care for have? Please tick all that apply.

- An autism spectrum disorder
- Asthma
- Arthritis
- Alcohol or substance misuse
- Coronary heart disease/heart failure
- Chronic Obstructive Pulmonary Disease (COPD)
- Cancer
- Dementia
- Diabetes
- A learning disability
- A mental health condition
- Needs that arise from being older (e.g. support with mobility)
- Another neurological condition (e.g Parkinson's, Motor Neurone Disease or MS)
- A physical disability
- They require palliative or end of life care
- A sensory impairment
- Stroke
- Other long term health condition
- Other (please specify)
- A combination of conditions or disabilities
- Prefer not to say

If you selected "other" or you would like to share further details of the health conditions the person that you care for has, please write them here

_______________________________________________________________________________

_______________________________________________________________________________

_______________________________________________________________________________

_______________________________________________________________________________

_______________________________________________________________________________

The person that I care for has problems with the following

- Blind or sight loss
- Deaf or hearing loss
- Speech impairment
- Precise hand movements
- Other
- Prefer not to say

If you selected "other" or you would like to share further details of the health problems the person that you care for has, please write them here

_______________________________________________________________________________

_______________________________________________________________________________

_______________________________________________________________________________

**If you care for another person, please fill the next section, otherwise please continue to Part 3.2**

**PERSON 3 that you care for**

How old is the person that you care for in years? Please select one.

- 0 to 15
- 16 to 24
- 25 to 34
- 35 to 44
- 45 to 54
- 55 to 64
- 65 to 74
- 75 to 84
- 85+
- Prefer not to say

What is the gender of the person that you care for? Please select one.

- Male
- Female
- Other
- Prefer not to say

What is the ethnicity of the person that you care for? Please select one.

- White
- Mixed/multiple ethnic groups
- Asian/Asian British
- Black/African/Caribbean/Black British
- Other ethnic group
- Prefer not to say

What condition(s) does the person that you care for have? Please tick all that apply.

- An autism spectrum disorder
- Asthma
- Arthritis
- Alcohol or substance misuse
- Coronary heart disease/heart failure
- Chronic Obstructive Pulmonary Disease (COPD)
- Cancer
- Dementia
- Diabetes
- A learning disability
- A mental health condition
- Needs that arise from being older (e.g. support with mobility)
- Another neurological condition (e.g Parkinson's, Motor Neurone Disease or MS)
- A physical disability
- They require palliative or end of life care
- A sensory impairment
- Stroke
- Other long term health condition
- Other (please specify)
- A combination of conditions or disabilities
- Prefer not to say

If you selected "other" or you would like to share further details of the health conditions the person that you care for has, please write them here

_______________________________________________________________________________

_______________________________________________________________________________

_______________________________________________________________________________

_______________________________________________________________________________

_______________________________________________________________________________

The person that I care for has problems with the following

- Blind or sight loss
- Deaf or hearing loss
- Speech impairment
- Precise hand movements
- Other
- Prefer not to say

If you selected "other" or you would like to share further details of the health problems the person that you care for has, please write them here

_______________________________________________________________________________

**If you care for another person, please fill the next section, otherwise please continue to Part 3.2**

**PERSON 4 that you care for**

How old is the person that you care for in years? Please select one.

- 0 to 15
- 16 to 24
- 25 to 34
- 35 to 44
- 45 to 54
- 55 to 64
- 65 to 74
- 75 to 84
- 85+
- Prefer not to say

What is the gender of the person that you care for? Please select one.

- Male
- Female
- Other
- Prefer not to say

What is the ethnicity of the person that you care for? Please select one.

- White
- Mixed/multiple ethnic groups
- Asian/Asian British
- Black/African/Caribbean/Black British
- Other ethnic group
- Prefer not to say

What condition(s) does the person that you care for have? Please tick all that apply.

- An autism spectrum disorder
- Asthma
- Arthritis
- Alcohol or substance misuse
- Coronary heart disease/heart failure
- Chronic Obstructive Pulmonary Disease (COPD)
- Cancer
- Dementia
- Diabetes
- A learning disability
- A mental health condition
- Needs that arise from being older (e.g. support with mobility)
- Another neurological condition (e.g Parkinson's, Motor Neurone Disease or MS)
- A physical disability
- They require palliative or end of life care
- A sensory impairment
- Stroke
- Other long term health condition
- Other (please specify)
- A combination of conditions or disabilities
- Prefer not to say

If you selected "other" or you would like to share further details of the health conditions the person that you care for has, please write them here

_______________________________________________________________________________

_______________________________________________________________________________

_______________________________________________________________________________

_______________________________________________________________________________

_______________________________________________________________________________

The person that I care for has problems with the following

- Blind or sight loss
- Deaf or hearing loss
- Speech impairment
- Precise hand movements
- Other
- Prefer not to say

If you selected "other" or you would like to share further details of the health problems the person that you care for has, please write them here

**Please now continue to Part 3.2**
