# Supplementary material for: Understanding Current Needs and Future Expectations of Informal Caregivers for Technology to Support Health and Well-being: National Survey Study
Source: JMIR Aging. 2022 Jan 27;5(1):e15413. doi: 10.2196/15413 (PMC8832269; doi:10.2196/15413)
Supplement: Multimedia Appendix 2 [file aging_v5i1e15413_app2.docx]

**Multimedia Appendix 2.** The number of different health conditions cared for across the sample.

| Dementia | 109 | (31) |
| --- | --- | --- |
| Needs that arise from being older (e.g. support with mobility) | 106 | (30) |
| A mental health condition | 74 | (21) |
| A combination of conditions or disabilities | 68 | (19) |
| A physical disability | 62 | (17) |
| An autism spectrum disorder | 60 | (17) |
| Arthritis | 55 | (15) |
| A learning disability | 52 | (15) |
| Other long term health condition | 52 | (15) |
| Neurological condition | 39 | (11) |
| Diabetes | 38 | (11) |
| A sensory impairment | 35 | (10) |
| Other (please specify) | 34 | (10) |
| Coronary heart disease/heart failure | 33 | (9) |
| Stroke | 28 | (8) |
| Cancer | 21 | (6) |
| Chronic Obstructive Pulmonary Disease (COPD) | 21 | (6) |
| Asthma | 15 | (4) |
| They require palliative or end of life care | 11 | (3) |
| Alcohol or substance misuse | 9 | (3) |
| Prefer not to say | 3 | (1) |
